# Corrigendum

**DOI:** 10.1002/osp4.386

**Published:** 2020-04-14

In the published article by Whitley et al,^1^ an author has encountered a problem with the search function in PubMed due to the fact that the author's full name is not included for the article. To resolve this, the author byline should read:

Jessica Cruz Whitley^1^, Carmen A. Peralta^1^, Mary Haan^1^, Allison E. Aiello^2^, Anne Lee^1^, Julia Ward^2^, Adina Zeki Al Hazzouri^3^, John Neuhaus^1^, Sally Moyce^4^ and Lenny López^1^

These changes have been implemented in the online version of the article.
